# CX3CR1 knockout aggravates Coxsackievirus B3-induced myocarditis

**DOI:** 10.1371/journal.pone.0182643

**Published:** 2017-08-11

**Authors:** Irene Müller, Kathleen Pappritz, Konstantinos Savvatis, Kerstin Puhl, Fengquan Dong, Muhammad El-Shafeey, Nazha Hamdani, Isabell Hamann, Michel Noutsias, Carmen Infante-Duarte, Wolfgang A. Linke, Sophie Van Linthout, Carsten Tschöpe

**Affiliations:** 1 Charité –Universitätsmedizin Berlin, corporate member of Freie Universität Berlin, Humboldt-Universität zu Berlin, and Berlin Institute of Health, Department of Internal Medicine and Cardiology, Campus Virchow Klinikum, Berlin, Germany; 2 DZHK (German Center for Cardiovascular Research), partner site Berlin, Germany; 3 Charité –Universitätsmedizin Berlin, corporate member of Freie Universität Berlin, Humboldt-Universität zu Berlin, and Berlin Institute of Health, Berlin-Brandenburg Center for Regenerative Therapies, Campus Virchow Klinikum, Berlin, Germany; 4 Inherited Cardiovascular Diseases Unit, Barts Health NHS Trust, Barts Heart Centre, London, United Kingdom; 5 William Harvey Research Institute, Queen Mary University London, London, United Kingdom; 6 Department of Cardiovascular Physiology, Ruhr University Bochum, Bochum, Germany; 7 Charité –Universitätsmedizin Berlin, corporate member of Freie Universität Berlin, Humboldt-Universität zu Berlin, and Berlin Institute of Health, Institute for Medical Immunology, Campus Virchow Klinikum, Berlin, Germany; 8 Department of Internal Medicine III, Division of Cardiology, Angiology and Intensive Medical Care, University Hospital Halle, Halle (Saale), Germany; Albert Einstein College of Medicine, UNITED STATES

## Abstract

Studies on inflammatory disorders elucidated the pivotal role of the CX3CL1/CX3CR1 axis with respect to the pathophysiology and diseases progression. Coxsackievirus B3 (CVB3)-induced myocarditis is associated with severe cardiac inflammation, which may progress to heart failure. We therefore investigated the influence of CX3CR1 ablation in the model of acute myocarditis, which was induced by inoculation with 5x10^5^ plaque forming units of CVB3 (Nancy strain) in either CX3CR1^-/-^ or C57BL6/j (WT) mice. Seven days after infection, myocardial inflammation, remodeling, and titin expression and phosphorylation were examined by immunohistochemistry, real-time PCR and Pro-Q diamond stain. Cardiac function was assessed by tip catheter. Compared to WT CVB3 mice, CX3CR1^-/-^ CVB3 mice exhibited enhanced left ventricular expression of inflammatory cytokines and chemokines, which was associated with an increase of immune cell infiltration/presence. This shift towards a pro-inflammatory immune response further resulted in increased cardiac fibrosis and cardiomyocyte apoptosis, which was reflected by an impaired cardiac function in CX3CR1^-/-^ CVB3 compared to WT CVB3 mice. These findings demonstrate a cardioprotective role of CX3CR1 in CVB3-infected mice and indicate the relevance of the CX3CL1/CX3CR1 system in CVB3-induced myocarditis.

## Introduction

Viral myocarditis is a cardiac disorder characterized by cardiac inflammation, which is often caused by cardiotropic viruses like Coxsackievirus group B type 3 (CVB3) and can progress to dilated cardiomyopathy (DCM) and congestive heart failure [1]. With respect to CVB3, a direct cardiomyocyte injury and subsequent long-term inflammatory reaction belong to the discussed mechanisms involved in the CVB3-induced pathogenesis [2,3]. However, the exact pathogenesis of CVB3-induced myocarditis needs still a better understanding [4] to find efficient therapeutic options counteracting the virus-induced inflammatory response [5].

Chemokine-induced migration of inflammatory cells plays a crucial role during cardiac inflammation [6–8]. Among the chemokine super-family, fractalkine (CX3CL1) exists in two distinct forms. The membrane-bound form serves as an adhesion protein. The soluble molecule has chemoattractant properties and is proteolytically cleaved from the cell membrane-anchored form of fractalkine [9,10]. Cellular sources of CX3CL1 include endothelial cells, epithelial cells, dendritic cells, macrophages and cardiomyocytes [9,11]. Both chemotaxis and adhesion are mediated by the G-protein-coupled receptor CX3CR1 [12], which is mainly expressed on natural killer cells, some T cell populations, dendritic cells and monocytes [10]. Interestingly, soluble CX3CL1 attracts natural killer cells, T cells, and dendritic cells and inhibits the function of the monocyte chemoattractant protein-1 (MCP-1) [10,13].

In previous studies, the CX3CL1/CX3CR1 system has been shown to be involved in the pathophysiology of cardiovascular disorders including heart failure [14,15] and inflammatory cardiomyopathy [9]. CX3CL1/CX3CR1 is also of relevance in the pathogenesis of other inflammatory disorders such as glomerulonephritis [16], rheumatoid arthritis [17] and cardiac allograft rejection [18]. Fractalkine and its receptor CX3CR1 have been shown to exert detrimental effects, since neutralization of the chemokine improved cardiac function after myocardial infarction [19] and inhibition of the respective receptor reduced atherosclerosis in mice [20]. Besides these findings, there are also data indicating a protective role of CX3CR1 since the loss of this receptor results in higher liver fibrosis in a model of hepatic fibrosis [21] and increased accumulation of inflammatory monocytes in gliomagenesis [22]. Abovementioned findings indicate the complexity of the CX3CL1/CX3CR1 system.

Since CX3CR1 is involved in inflammatory disorders, cardiovascular diseases and viral infection, and due to the lack of data regarding the role of CX3CR1 in viral experimental myocarditis, we aimed to investigate the pathophysiological role of CX3CR1 in experimental CVB3-induced acute myocarditis.

## Materials and methods

### Induction of myocarditis and hemodynamic measurements

Six week-old male C57BL6/j mice (further named as WT; provided by the Forschungseinrichtung für experimentelle Medizin (FEM), Berlin, Germany) and CX3CR1 deficient mice (further named as CX3CR1^-/-^, provided by I. Hamann and C. Infante-Duarte [23]) were randomly divided into 4 groups (n = 7–12 per group). The experimental groups consisted of WT, WT CVB3, CX3CR1^-/-^, and CX3CR1^-/-^ CVB3. Mice were either treated with 5×10^5^ plaque forming units of CVB3 (Nancy strain) or with saline. One week after infection, all animals were anesthetized (Urethane 0.8–1.2 g/kg i.p., Sigma, Germany; Buprenorphin 0.05 mg/kg i.p. Essex Pharma, Germany), intubated, and artificially ventilated with a rodent ventilator type 7025 (Ugo Basile, Comerio VA, Italy). Via a tip catheter (1.2F) system, indices of systolic and diastolic left ventricular function were recorded as described previously [2,24]. After determination of the cardiac function, mice were euthanized by cervical dislocation and the left ventricle (LV) and spleen were excised, immediately snap frozen in liquid nitrogen and stored at -80°C for molecular biology and immunohistological analyses. This investigation conforms to the European principles of laboratory animal care (Directive 2010/63/EU) and was approved by the local ethical committee (Landesamt für Gesundheit und Soziales, Berlin, Nr: G0313/09). Mice were kept under standard conditions (12∶12 h light-dark cycle, 20–24°C), with unlimited access to water and food. After CVB3 infection, mice were daily monitored and scored according the following criteria: 0 = no disease; 1 = weight loss; 2 = reduced activity; 3 = apathy and severe signs of heart failure.

### RNA isolation, cDNA synthesis and gene expression analysis

For total RNA isolation from murine tissue, the TRIzol^®^ (Life Technologies GmbH, Darmstadt, Germany) method was used followed by cDNA synthesis with the High Capacity Kit (Life Technologies GmbH). To assess the relative mRNA expression of the target genes in murine LVs and spleens, real-time PCR was performed on a QuantStudioTM 6 Flex (Life Technologies) using gene expression assays from Life Technologies (Table 1). All data were normalized to the housekeeping genes 18S as endogenous controls (Table 1) and are expressed using the 2^-ΔCt^ formula followed by a normalization to the WT group, which was set as 1. CVB3 mRNA expression was detected using the forward primer and the reverse primer at a final concentration of 60 ng/μl and a FAM-labelled MGB probe at a final concentration of 5 pM (TIB Molbiol, Berlin Germany; Table 1).

**Table 1:** Reporter assays and primers.

Gene		Assay number
Monocyte chemotactic Protein-1	MCP-1	Mm00441242_m1
C-C chemokine receptor type 2	CCR2	Mm00438270_m1
chemokine (C-X3-C motif) ligand 1	CX3CL1	Mm00436454_m1
chemokine (C-X3-C motif) receptor 1	CX3CR1	Mm02620111_s1
	Ly6C	Mm03009946_m1
intercellular cell adhesion molecular-1	ICAM-1	Mm00516023_m1
vascular cell adhesion molecular-1	VCAM-1	Mm01320970_m1
Tumor necrosis factor α	TNF-α	Mm00443258_m1
Interferon γ	IFN-γ	Mm00801778_m1
Interleukin 1β	IL-1β	Mm00434228_m1
Interleukin 6	IL-6	Mm00446190_m1
Endothelial Nitric Oxide Synthase	iNOS	Mm00440502_m1
Inducible Nitric Oxide Synthase	eNOS	Mm01164908_m1
NADPH oxidase 1	NOX1	Mm00549170_m1
NADPH oxidase 4	NOX4	Mm00479246_m1
Transforming growth factor β1	TGF-β1	Mm00441724_m1
	Bax	Mm00432050_m1
B-cell lymphoma 2	Bcl-2	Mm00477631_m1
Interferon β	IFN-β	Mm00439546_s1
Housekeeping gene	18s	Hs99999901_s1
CVB3 forward primer		5´-CCCTGAATGCGGCTAATCC-3´m
CVB3 reverse primer		5´-ATTGTCACCATAAGCAGCCA-3´m
FAM-labeled MGB probe		FAM 5´-TGCAGCGGAACCG-3

### Immunohistological measurements

The obtained tissue samples of the LV were embedded in Tissue-Tek OCT compound (Sakura, Zoeterwoude, Netherlands). Immunohistochemistry was performed with specific antibodies directed against CX3CL1 (rabbit anti-CX3CL1, Abcam, Cambridge, United Kingdom), CD4 (rat anti-CD4, BD Biosciences, Heidelberg, Germany), CD3 (rat anti-CD3, BD Biosciences), CD68 (rabbit anti-CD68, Abcam), ICAM-1 (armenian hamster anti-ICAM-1, BD Biosciences), VCAM (rat anti-VCAM, BD Biosciences) collagen I (rabbit anti-Col I, Chemicon, Darmstadt, Germany), and collagen III (rabbit anti-Col III, Calbiochem, Darmstadt, Germany). Quantification was performed by digital image analyses using a 200x magnification, as described in detail elsewhere [25]. For the identification of specific CX3CL1-expressing cells, stainings were performed on successive Tissue-Tek OCT embedded tissue samples by using the CX3CL1 antibody as mentioned above and cell-specific markers including CD68 (rabbit anti-CD68, Abcam), CD11b (rat anti-CD11b, BD Biosciences), Ly6g (rat anti-Ly6g, GeneTex, Irvine, USA), CD4 (rat anti-CD4, BD Biosciences), CD8a (rat anti-CD8a, Biolegend), and fibroblast-marker (rat anti-fibroblast, Santa Cruz, Heidelberg, Germany). Overall, congruent areas were identified on the successive CX3CL1 and respective cell marker slides. Subsequently, digital images at a 200x magnification were taken for representative pictures.

### All-titin phosphorylation by Pro-Q Diamond stain

The composition as well as the phosphorylation state of the cardiac titin isoform N2B was determined using Pro-Q Diamond phospho-protein stain as described elsewhere [26]. To preserve the endogenous phosphorylation state of the proteins, frozen tissues from LV mouse hearts were solubilized and treated as described for titin isoform separation. The gels were stained for 1 h with Pro-Q Diamond phosphoprotein stain. Fixation, washing and de-staining were performed according to the manufacturer’s guidelines. To assess total protein content, gels were stained overnight with SYPRO Ruby. Staining was visualized using the LAS-4000 Image Reader (460 nm/605 nm Ex/Em; 2 s illumination) and signals were analyzed with Multi Gauge V3.2 and AIDA software.

### Statistical analysis

Statistical analysis was performed using GraphPad Prism 5.0 software (GraphPad Software, La Jolla, CA). Data are expressed as the mean ± SEM. Ordinary one-way ANOVA was performed to compare parametric data, whereas non-parametric data were compared with Kruskal-Wallis. Both analyses were followed by a post hoc test. Differences were considered statistically significant at a value of p<0.05.

## Results

### Impact of CX3CR1^-/-^ on chemokine receptor and chemokine expression in Coxsackievirus B3-infected mice

**Fig 1:**
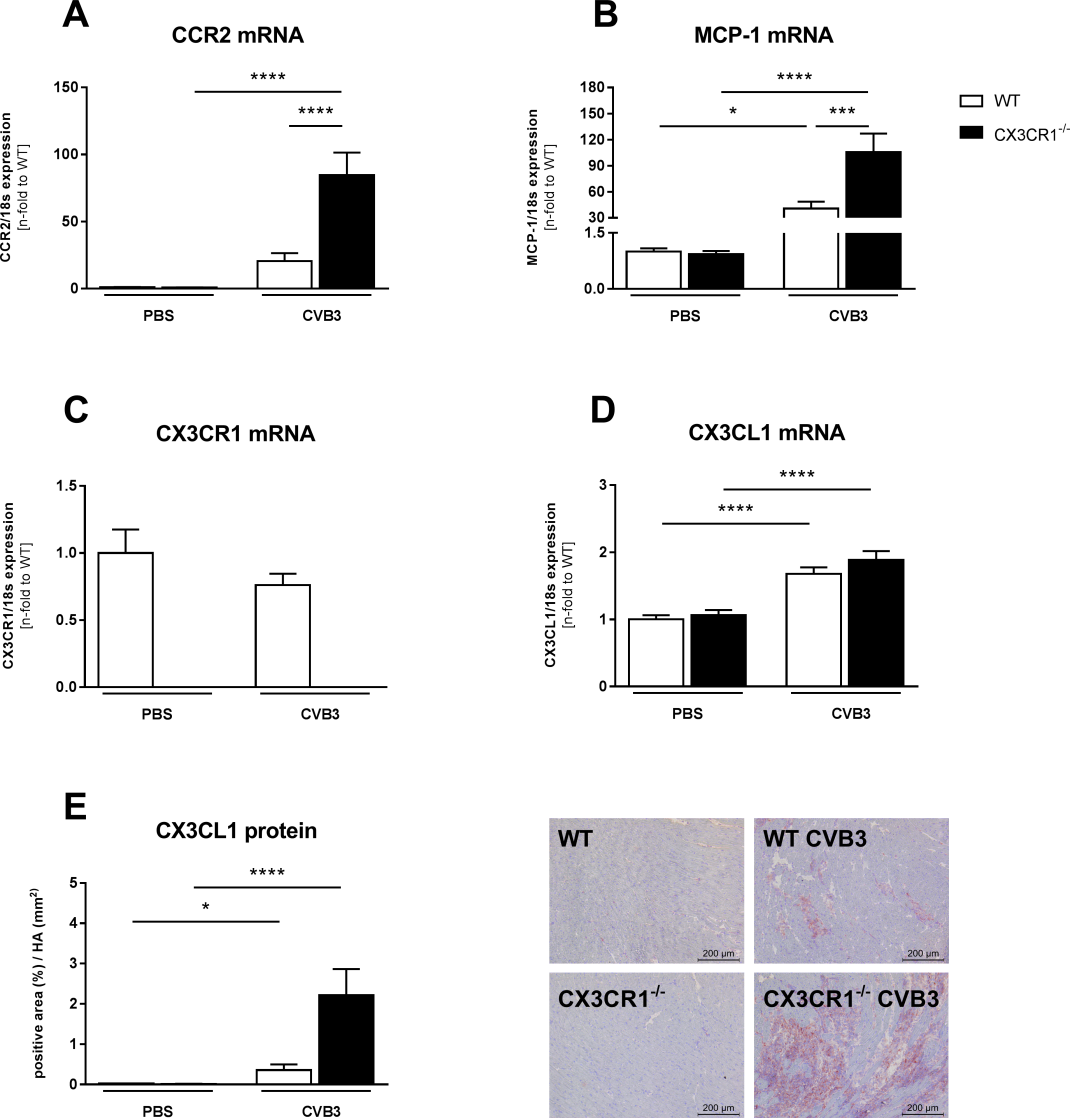
Impact of CX3CR1^-/-^ on left ventricular chemokine receptor and chemokine expression in Coxsackievirus B3-infected mice. Bar graphs represent the mean ± SEM of gene expression data of LV (A) CCR2, (B) MCP-1, (C) CX3CR1, and (D) CX3CL1, as indicated, after normalization to the housekeeping gene 18S using the 2−^Δ^Ct formula and normalized to the WT group, which was set as 1. Quantitative CX3CL1 protein expression (E) was determined via digital image analysis at a 200x magnification (scale bar = 200 μm). Bar graphs represent the mean ± SEM. Statistical analysis was performed by One-way ANOVA or the Kruskal-Wallis test. *p<0.05, ***p<0.001, ****p<0.0001 with n = 7–12 per group.

Since the CX3CR1/CX3CL1 system is upregulated in inflammatory cardiomyopathy and has chemotactic properties [9], we examined the impact of CX3CR1^-/-^ on cardiac chemokine receptor and chemokine expression in CVB3-induced myocarditis. LV CCR2 mRNA levels were slightly upregulated in WT CVB3-infected mice versus control mice, which was accompanied by a 41-fold (p<0.05) increase of LV MCP-1 levels (Fig 1A and 1B). Ablation of CX3CR1 led to 4.1-fold (p<0.0001) and 2.6-fold (p<0.001) increased CCR2 and MCP-1 mRNA levels in CX3CR1^-/-^ CVB3 vs WT CVB3 mice, respectively (Fig 1A and 1B). LV CX3CR1 expression was unaltered in WT CVB3-infected versus control mice (Fig 1C). Additionally, CX3CL1 gene expression was 1.7-fold (p<0.0001) induced in WT CVB3 mice versus control mice (Fig 1D), which was paralleled by a 17-fold (p<0.05) enhanced LV CX3CL1 protein expression in those mice (Fig 1E). CX3CR1^-/-^ CVB3 mice exhibited no significant changes of LV CX3CL1 gene and protein levels versus WT CVB3 mice. Detailed analysis of LV CX3CL1 expression by immunohistochemistry demonstrated that CX3CL1 is expressed by infiltrated CD11b, CD68, Ly6g+ cells, and cardiac fibroblasts, but not by CD4 and CD8 cells (S1 Fig).

In parallel to the chemokine regulation in the LV, splenic CCR2, MCP-1, and CX3CR1 gene expression was 2.2-fold (p<0.05), 3.5-fold (p<0.001), and 1.6-fold (p<0.05) increased in CVB3-infected compared to control mice, respectively (S2A-S2C Fig). CCR2 mRNA levels were further 3.2-fold (p<0.05) enhanced in the spleen of CX3CR1^-/-^ CVB3 vs WT CVB3 mice (S2A Fig).

### Impact of CX3CR1^-/-^ on cardiac inflammatory cell presence, adhesion molecules, and cytokines in Coxsackievirus B3-infected mice

**Fig 2:**
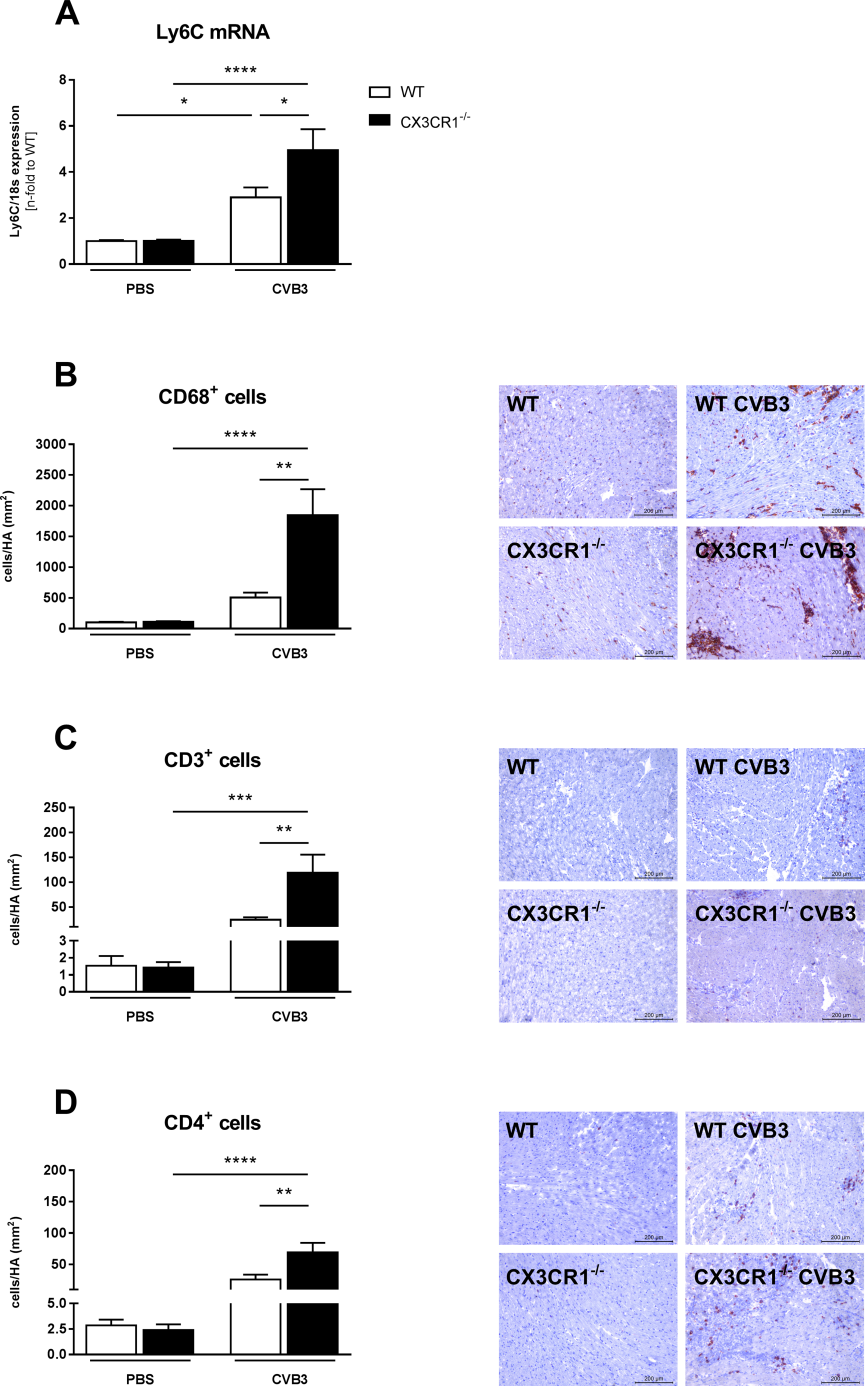
CX3CR1^-/-^ induces left ventricular cell infiltration in Coxsackievirus B3-infected mice. (A,B) Gene expression level of Ly6C and immunohistological staining of CD68^+^ cells as marker for monocyte infiltration (C,D) Staining for CD3^+^ and CD4^+^ cells in the myocardium after CVB3-infection. The quantitative determination of the immune staining was performed via digital image analysis at a 200x magnification (scale bar = 200 μm). Bar graphs represent the mean ± SEM. Statistical analysis was performed by One-way ANOVA or the Kruskal-Wallis test. *p<0.05, **p<0.01, ***p<0.001, ****p<0.0001 with n = 7–12 per group.

**Fig 3:**
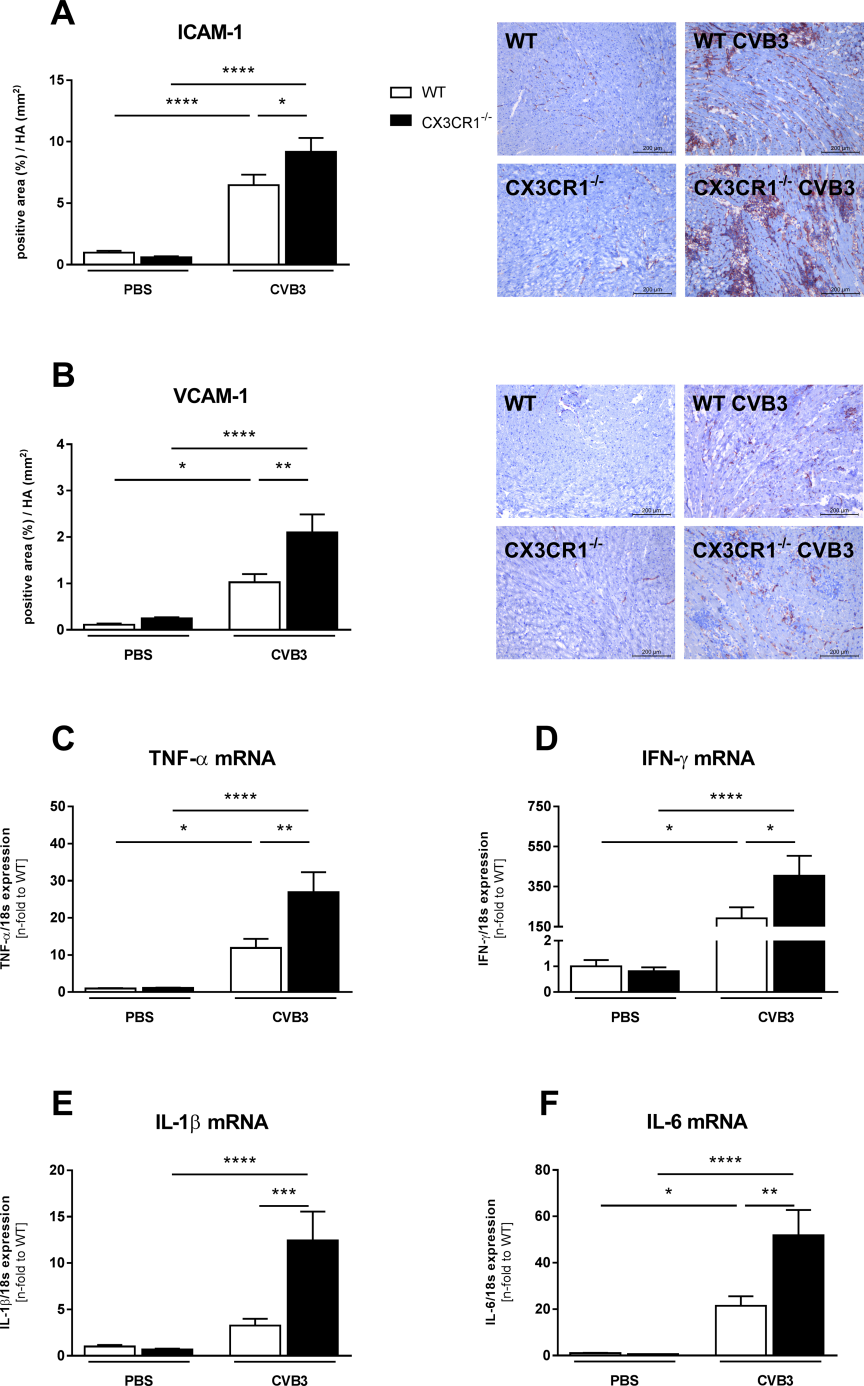
CX3CR1^-/-^ enhances left ventricular expression of adhesion molecules and pro-inflammatory cytokines in Coxsackievirus B3-infected mice. Induction of ICAM-1 (A) and VCAM-1 (B) as well as of pro-inflammatory cytokines (C-F) in CX3CR1^-/-^ mice compared to WT CVB3 mice. The quantitative determination of ICAM-1 and VCAM-1, indicated by the positive area (%) in relation to the heart area (mm^2^), was performed via digital image analysis at a 200x magnification (scale bar = 200 μm). Bar graphs represent the mean ± SEM. Statistical analysis was performed by One-way ANOVA or the Kruskal-Wallis test. *p<0.05, **p<0.01, ***p<0.001, ****p<0.0001 with n = 7–12 per group.

The upregulated LV MCP-1 expression in CX3CR1^-/-^ CVB3 versus WT CVB3 mice was accompanied by a 1.7-fold (p<0.05), 3.6-fold (p<0.01), 4.9-fold (p<0.01), and 2.8-fold (p<0.01) increase of LV Ly6C mRNA levels and CD68^+^, CD3^+^ and CD4^+^ cells, respectively (Fig 2). In parallel to the enhanced presence of inflammatory cells, CX3CR1^-/-^ CVB3 animals exhibited 1.4-fold (p<0.05) and 2.1-fold (p<0.01) increased expression levels of intercellular adhesion molecular-1 (ICAM-1) and vascular cell adhesion molecular-1 (VCAM-1), respectively (Fig 3A and 3B). The increased infiltration of immune cells in CX3CR1^-/-^ CVB3 compared to WT CVB3 mice was further reflected in 2.3-fold (p<0.01), 2.1-fold (p<0.05), 2.4-fold (p<0.01) and 3.8-fold (p<0.001) higher mRNA expression of the CVB3-induced pro-inflammatory cytokines TNF-α, IFN-γ, IL-1β, and IL-6, respectively (Fig 3C–3F).

### Impact of CX3CR1^-/-^ on oxidative stress in Coxsackievirus B3-infected mice

**Fig 4:**
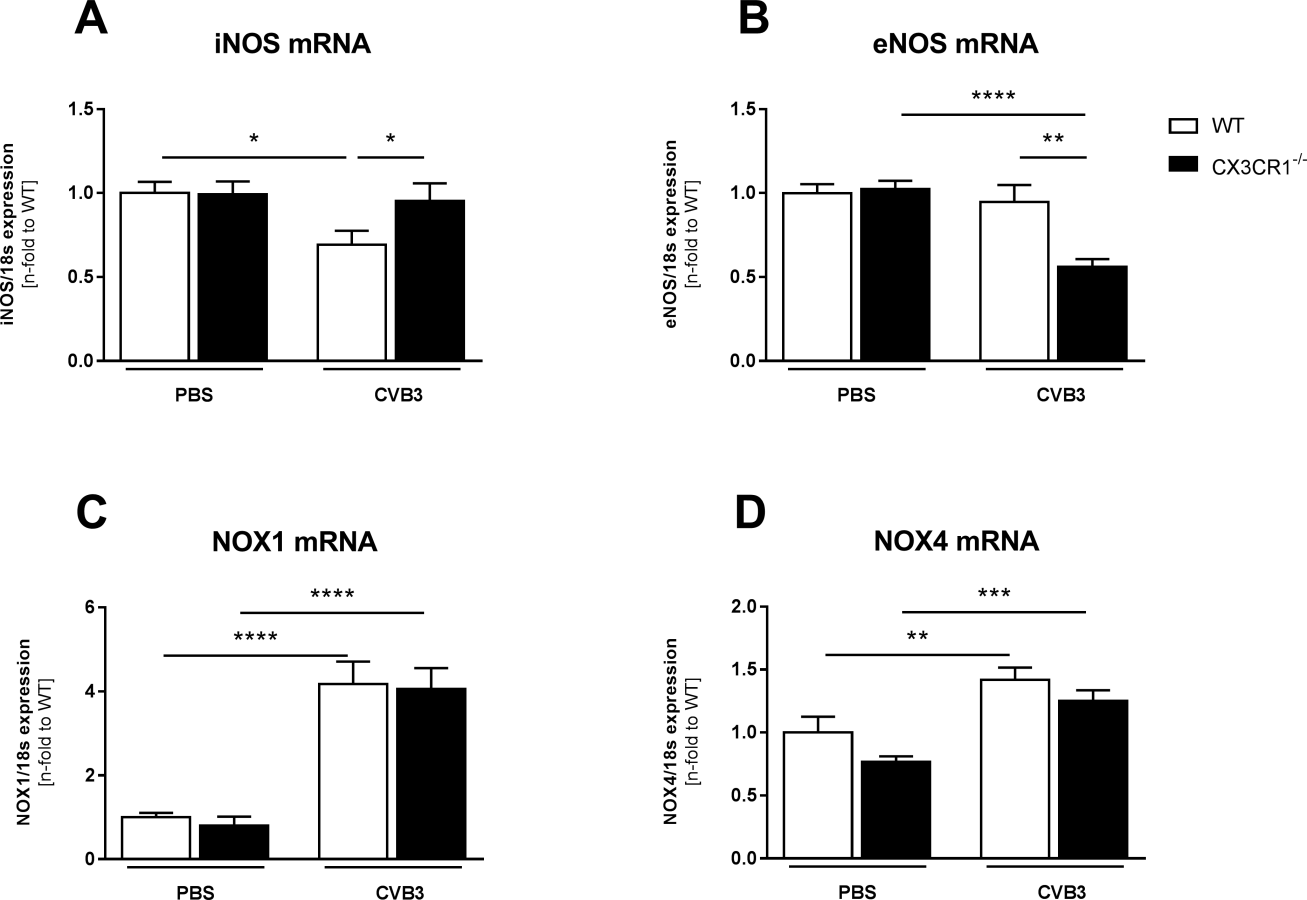
CX3CR1^-/-^ displays increased left ventricular oxidative stress in Coxsackievirus B3-infected mice. Bar graphs represent the mean ± SEM of gene expression data of LV (A) iNOS (B) eNOS, (C) NOX11, and (D) NOX4, as indicated, after normalization to the housekeeping gene 18S using the 2−^Δ^Ct formula and normalized to the WT group, which was set as 1. (A,B) Gene expression analysis of NOS show differences between CX3CR1^-/-^ CVB3 and WT CVB3 mice. Additional, no changes in NOX mRNA expression between CX3CR1^-/-^ CVB3 and WT CVB3 mice could be observed (C,D). Statistical analysis was performed by One-way ANOVA or the Kruskal-Wallis test. *p<0.05, **p<0.01, ***p<0.001, ****p<0.0001 with n = 7–12 per group.

CX3CR1^-/-^ CVB3 mice exhibited 1.4-fold (p<0.05) higher LV iNOS mRNA levels compared to WT CVB3 mice (Fig 4A). In contrast, CX3CR1^-/-^ CVB3 mice displayed 1.7-fold (p<0.01) lower eNOS mRNA levels vs WT CVB3 mice (Fig 4B). Interestingly, neither NOX1 nor NOX4 mRNA levels were changed when comparing CX3CR1^-/-^ CVB3 vs WT CVB3 mice (Fig 4C and 4D).

### Impact of CX3CR1^-/-^ on cardiac fibrosis and titin phosphorylation in Coxsackievirus B3-infected mice

**Fig 5:**
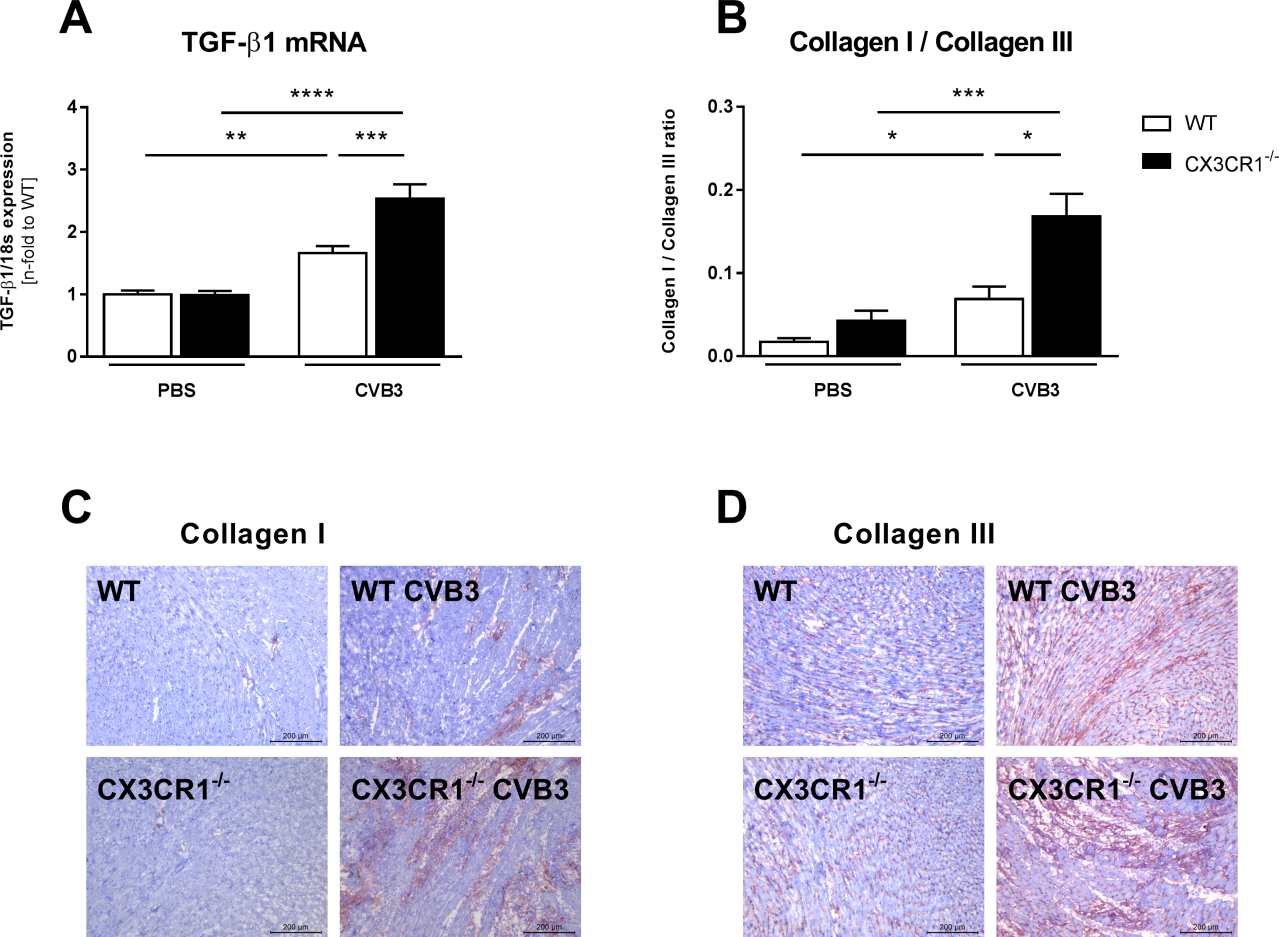
CX3CR1^-/-^ leads to enhancement of myocardial fibrosis in Coxsackievirus B3-infected mice. Bar graphs represent the mean ± SEM of LV (A) TGF-ß1 gene expression, as indicated, after normalization to the housekeeping gene 18S using the 2−^Δ^Ct formula and normalized to the WT group, which was set as 1, and (B) collagen I/III ratio, demonstrated by immunohistological stainings of (C) collagen I and (D) collagen III. Specific epitopes of collagen I and collagen III are coloured red as shown by representative images (scale bar = 200 μm). Quantification of the positive area (%) / heart area (mm^2^) was performed via digital image analysis. Statistical analysis was performed by One-way ANOVA or the Kruskal-Wallis test. *p<0.05, **p<0.01, ***p<0.001, ****p<0.0001 with n = 7–12 per group.

**Fig 6:**
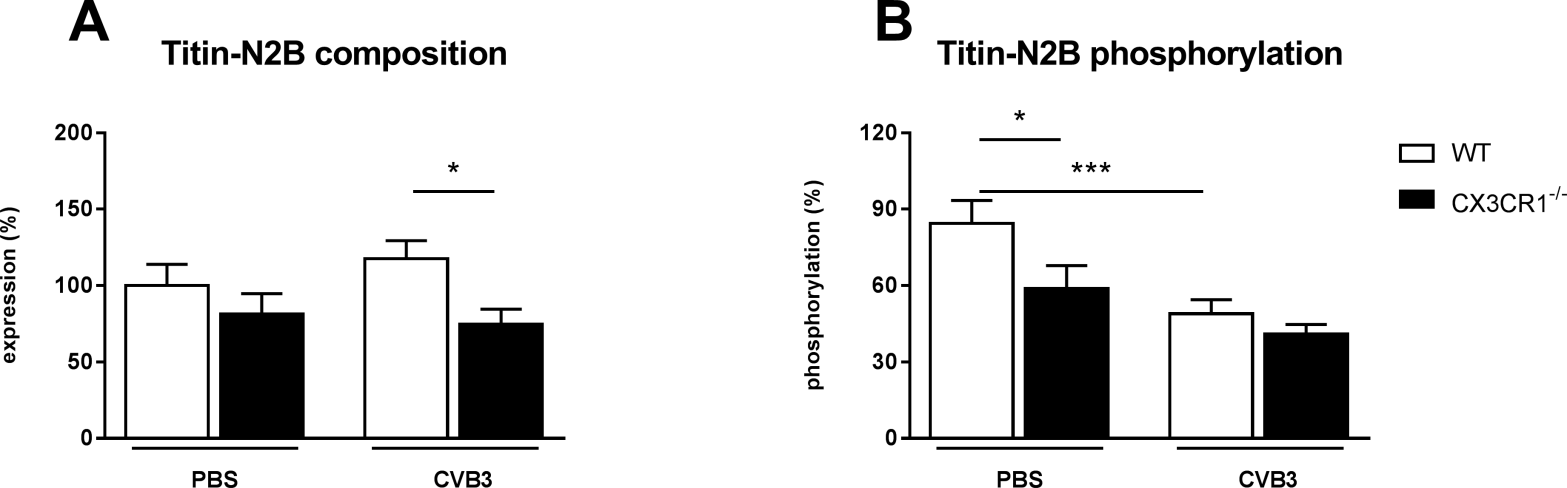
CX3CR1^-/-^ results in changes in titin isoform phosphorylation. (A) Expression of the N2B isoform and the corresponding phosphorylation (B). The composition as well as the phosphorylation state of the cardiac titin isoform N2B was determined using Pro-Q Diamond phospho-protein stain. In contrast to N2B phosphorylation the cardiac titin isoform composition is different in CX3CR1^-/-^ CVB3 mice to WT CVB3 mice, respectively. Bar graphs represent the mean ± SEM. Statistical analysis was performed by One-way ANOVA or the Kruskal-Wallis test. *p<0.05, ***p<0.001 with n = 7–12 per group.

Evaluation of the expression of the pro-fibrotic factor TGF-β1 [27] revealed that CX3CR1^-/-^ CVB3 mice displayed 1.5-fold (p<0.001) higher LV TGF-β1 mRNA levels versus WT CVB3 mice (Fig 5A). In agreement, the collagen I / III protein ratio was 2.4-fold (p<0.05) higher in CX3CR1^-/-^ CVB3 mice compared to WT CVB3 mice, indicating an elevated pathological collagen deposition (Fig 5B). Since besides cardiac fibrosis also titin dysregulation [28] contributes to left ventricular dysfunction, we next evaluated the impact of CX3CR1^-/-^ on the titin N2B isoform composition and phosphorylation state. CX3CR1^-/-^ CVB3 mice exhibited 1.5-fold (p<0.05) lower titin N2B expression levels compared to WT CVB3 mice (Fig 6A). Titin phosphorylation was 1.6-fold (p<0.001) reduced in WT CVB3 compared to uninfected controls. A 1.4-fold (p<0.05) decrease in titin phosphorylation was also present in CX3CR1^-/-^ vs WT mice (Fig 6B).

### Impact of CX3CR1^-/-^ on cardiac apoptosis, IFN-β expression, and CVB3 presence in Coxsackievirus B3-infected mice

**Fig 7:**
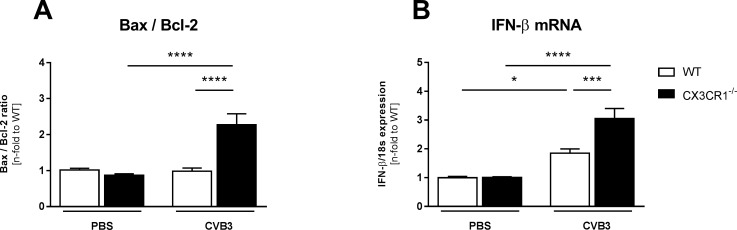
CX3CR1^-/-^ increases apoptosis and anti-viral cytokine expression in Coxsackievirus B3-infected mice. (A) The Bax/Bcl-2 ratio as index for cardiomyocyte apoptosis indicates increased cardiomyocyte apoptosis in infected CX3CR1^-/-^ mice compared to the corresponding WT mice. (B) Gene expression of anti-viral IFN-β is increased in CX3CR1^-/-^ CVB3 versus WT CVB3 mice. Bar graphs represent the mean ± SEM. Statistical analysis was performed by One-way ANOVA or the Kruskal-Wallis test. *p<0.05, ***p<0.001, ****p<0.0001 with n = 7–12 per group.

Since cardiomyocyte apoptosis is an important trigger for CVB3 viral progeny release [2], we next analyzed the impact of CX3CR1^-/-^ on a marker of cardiomyocyte apoptosis, the Bax/Blc2 ratio [29–32]. CX3CR1^-/-^ CVB3 mice displayed a 2.1-fold (p<0.0001) higher Bax/Bcl2 ratio compared to WT CVB3 mice (Fig 7A). In parallel to this enhanced ratio, the anti-viral cytokine IFN-β [33] was 1.6-fold (p<0.001) increased in CX3CR1^-/-^ CVB3 vs WT CVB3 mice (Fig 7B). CX3CR1^-/-^ CVB3 mice displayed 5.6-fold (p<0.05) higher CVB3 mRNA levels compared to WT CVB3 mice (WT CVB3: 5.4x10^4^ ± 1.0x10^4^ (n = 8) vs CX3CR1^-/-^ CVB3: 3.0x10^5^ ± 8.5x10^4^ (n = 10); p<0.05).

### Impact of CX3CR1^-/-^ on hemodynamic function in Coxsackievirus B3-infected mice

**Fig 8:**
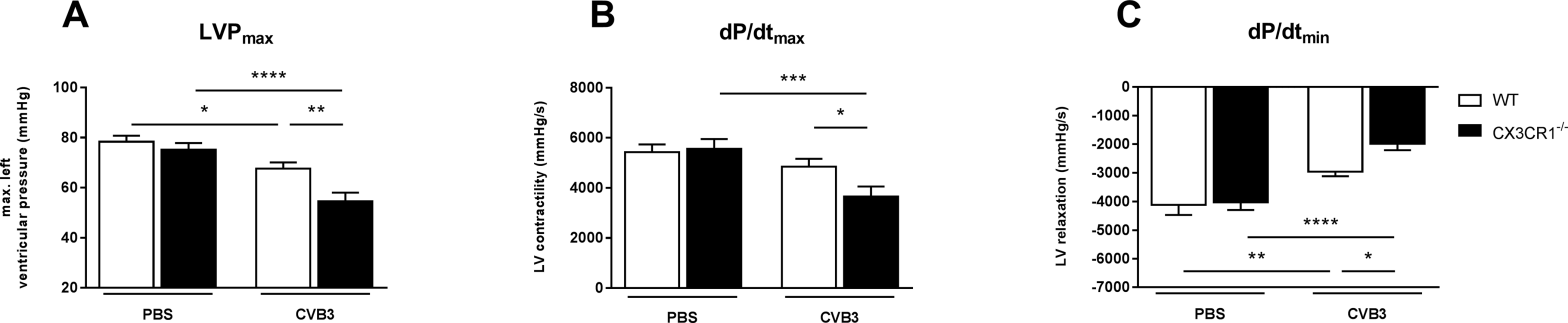
CX3CR1^-/-^ impaired left ventricular function in Coxsackievirus B3-infected mice. Evaluation of cardiac function via tip catheter. After inoculation with CVB3 CX3CR1^-/-^ mice show declined heart function as indicated by (A) reduced max. LV pressure (LVP_max_), (B) reduced LV contractility (dP/dt_max_) and (C) decreased LV relaxation (dP/dt_min_). Bar graphs represent the mean ± SEM. Statistical analysis was performed by One-way ANOVA or the Kruskal-Wallis test. *p<0.05, **p<0.01, ***p<0.001, ****p<0.0001 with n = 7–12 per group.

Given the impact of CX3CR1^-/-^ on cardiac inflammation, fibrosis, N2B titin, and apoptosis in CVB3-infected myocarditis mice, we next evaluated whether these effects were translated in changes in left ventricular function. Hemodynamic characterization demonstrated that CX3CR1^-/-^ CVB3 mice exhibited an impaired systolic and diastolic function compared to WT CVB3 mice as indicated by 19% (p<0.01), 25% (p<0.05) and 33% (p<0.05) impaired LVP_max_, dP/dt_max_, and dP/dt_min_, respectively (Fig 8).

## Discussion

In the present study, we could demonstrate that CX3CR1 plays a critical role in the pathogenesis of CVB3-induced myocarditis. This follows from the findings that CX3CR1^-/-^ CVB3 mice exhibited an exacerbated CVB3-induced myocarditis as shown by a higher LV MCP-1 and CCR2 expression, more cardiac infiltrates, and higher cytokine levels versus CVB3-infected WT mice. In addition, cardiac fibrosis, apoptosis and CVB3 presence were more pronounced and titin was dysregulated in CX3CR1^-/-^ CVB3 mice compared to WT CVB3 mice.

Although the exact pathogenesis of CVB3-induced myocarditis is still a matter of debate [4], chemokine-induced cardiac migration of inflammatory mononuclear cells is considered to be the initial step [7]. Nevertheless, the role of the chemokine receptor CX3CR1 has not been unraveled yet in experimental CVB3-induced myocarditis. Accumulating evidence demonstrates that the CX3CL1/CX3CR1 axis plays a role in cardiac disorders. CVB3-positive inflammatory cardiomyopathy patients showed induced CX3CL1 expression in endomyocardial biopsies and increased CX3CR1 expression in peripheral blood mononuclear cells [9]. This finding encouraged us to examine the role of CX3CR1 in CVB3-induced myocarditis.

In agreement with the abovementioned findings in CVB3-positive inflammatory cardiomyopathy patients [9], we observed increased CX3CL1 mRNA and protein expression levels in the LV of CVB3-infected versus control mice. Since CX3CL1 is expressed by CD68 and CD11b monocytes/macrophages and cardiac fibroblasts (S1 Fig), the upregulation in LV CX3CL1 expression in CVB3-infected mice can partly be explained by the increase in monocytes/macrophages (Fig 2) and cardiac fibroblast presence (Fig 5) in myocarditis mice. Conform to the systemic increased CX3CR1 expression in peripheral blood mononuclear cells of CVB3-positive inflammatory cardiomyopathy patients [9], an upregulation in CX3CR1 mRNA expression was found in the spleen of CVB3-infected myocarditis compared to control mice. However, CX3CR1 LV mRNA levels were unchanged among CVB3 and control mice. Furthermore, we showed an increased expression of the chemokine MCP-1 and its receptor CCR2 in cardiac and splenic tissue of CVB3-induced myocarditis versus control mice. This is in further harmony with the MCP-1 findings in EMBs and peripheral blood mononuclear cells of CVB3-positive inflammatory cardiomyopathy patients [9]. Both MCP-1 and CCR2 play a pivotal in the CVB3-induced myocarditis pathogenesis [7]. In addition, the expression of Ly6C-positive monocytes was also increased in the LVs of CVB3-infected mice. Interestingly, CX3CR1^-/-^ CVB3 mice exhibited higher LV MCP-1 and CCR2 expression levels, but no further increase in LV CX3CL1 expression compared to WT CVB3 mice. Furthermore, the increased LV MCP-1 and CCR-2 levels were also accompanied by enhanced Ly6C expression and a higher presence of CD68 positive cells. The data concerning LV MCP-1 and Ly6C are consistent with other findings where loss of CX3CR1 increased the accumulation of inflammatory monocytes [22], whereas the expression of CCR2 and CD68 positive cells in CX3CR1^-/-^ mice has not been elucidated before. Additionally, we observed an induction of the adhesion molecules VCAM-1 and ICAM-1 in the LV, which is in harmony with the increased levels of MCP-1 and CCR2, which are triggers of these two adhesion molecules [34].

Basically, these observations suggest that under experimental CVB3 conditions, the infiltration of pro-inflammatory monocytes dominates over the invasion of anti-inflammatory mononuclear cells, since it has been shown that the first subtype invades via the CCR2 and MCP-1 pathway, whereas anti-inflammatory monocytes invade in a CX3CR1-dependent manner [35,36]. Furthermore, CX3CR1 seems to control the CCR2/MCP-1-dependent pathway, since CX3CR1 ablation resulted in an exacerbation of CVB3-induced myocarditis. This control mechanism was also postulated by Menasria [35].

In harmony with the increased inflammatory cell presence in CX3CR1^-/-^ CVB3 versus WT CVB3 mice, we demonstrated enhanced LV cytokine expression including IL-6, IL-1β, TNF-α and IFN-γ in CX3CR1^-/-^ CVB3 compared to WT CVB3 mice. This is in alignment with other findings, where the loss of CX3CR1^-/-^ led to enhanced IL-6, IL-1β, TNF-α, and IFN-γ levels and where IL-1β is stated as a canonical inducer of MCP-1 [22,35]. Additionally, the enhanced presence of CD3+ and CD4+ lymphocytes in CX3CR1^-/-^ CVB3 mice may also account for the enhanced inflammatory mediator levels.

Besides the detrimental CVB3-induced inflammatory effect, also oxidative stress leads to an aggravation of myocardial injury [37] and thus exacerbates inflammation [38]. No changes were found in the expression of NOX1 and NOX4, which comprise two of the seven NADPH oxidases members [39] and are a major source of ROS [40]. However, iNOS and eNOS, the two main members of the NO family synthases [41], were differently expressed in CX3CR1^-/-^ CVB3 mice compared to WT CVB3 animals: whereas eNOS was downregulated in CX3CR1^-/-^ CVB3 versus WT CVB3 mice, iNOS expression was increased in those mice. This suggests a higher oxidative stress in the LV of CVB3 mice deficient for CX3CR1 compared to WT CVB3 mice, which might underlie the increased viral presence in CX3CR1^-/-^ CVB3 versus WT CVB3 mice since oxidative stress and subsequent cardiomyocyte apoptosis contribute to viral progeny release [2]. Along with this hypothesis, the Bax/Bcl2 ratio, an important marker for cardiomyocyte apoptosis as consistently shown by Spillmann *et al*. [29], Leri *et al*. [30,31], as well as by Van Linthout *et al*. [32], was higher in CX3CR1^-/-^ CVB3 mice compared to WT CVB3 mice.

The more pronounced cardiac fibrosis in CVB3-infected CX3CR1^-/-^ versus CVB3 WT mice, as reflected by an enhancement of pro-fibrotic TGF-β1 [27] and collagen I and III expression, is in line with mouse CX3CR1^-/-^ models of liver or kidney fibrosis [42,43]. Since TGF-β is produced by infiltrating monocytes and T cells [44,45], these findings suggest that the enhanced infiltration of monocytes and T cells in CX3CR1^-/-^ CVB3 mice underlie the raised expression of TGF-β, leading to more myofibroblasts and thus more collagen I and III in CX3CR1^-/-^ CVB3 versus WT CVB3 mice, further corroborating the link between cardiac inflammation and cardiac fibrosis [27,46].

The giant myofilament titin influences cardiac contractility and relaxation [28]. Two main titin isoforms exist: N2B and N2BA, of which among others impaired N2B phosphorylation leads to cardiac dysfunction [26]. The present study revealed no changes between WT CVB3 and CX3CR1^-/-^ CVB3 mice in the phosphorylation state of N2B, while we could demonstrate a lower N2B titin isoform expression in CX3CR1^-/-^ CVB3 mice. Mutations in the N2B isoform have been shown to be associated with DCM and hypertrophic CM in humans [47], and targeted deletion of the N2B region in mice resulted in diastolic dysfunction [48]. Based on these data, we assume that besides the accumulation of collagens, the lower N2B isoform expression might explain the reduced cardiac function, as displayed by a reduction of the systolic and diastolic parameters dP/dt_max_, and dP/dt_min_ in CX3CR1^-/-^ mice compared to the WT infected animals. A further explanation for the impaired cardiac function in CX3CR1^-/-^ CVB3 mice versus WT CVB3 mice might be the increased apoptosis of cardiomyocytes, as suggested by the higher Bax/Bcl-2 ratio in CX3CR1^-/-^ CVB3 versus WT CVB3 mice [29]. Overall, cardiac function was determined with a tip catheter. In contrast to a non-invasive method as echocardiography, this method only allows the measurement at a single time point, which is hence a limitation of the study.

## Conclusion

Our observations allow us to conclude that CX3CR1 emerges to be cardioprotective in the pathogenesis of CVB3-induced myocarditis since CX3CR1^-/-^ mice were more susceptible to CVB3 infection as WT animals. A functional CX3CR1 seems to be critical for the control of MCP-1/CCR2-mediated inflammatory cell invasion and the related inflammatory response, apoptosis, and CVB3 presence. Nevertheless, other studies demonstrated a controversial role of CX3CR1, which emphasizes the complexity of the CX3CL1/CX3CR1 system and its potential difference depending on the pathogenic disorder. Finally, we conclude that the modulation of the CX3CL1/CX3CR1 axis could be a new important target in the treatment of CVB3-induced myocarditis.

## Supporting information

S1 Fig CX3CL1-expressing cells in the left ventricle of mice. Representative pictures of immunohistological stainings on successive tissue samples from LV (A) CD68+ cells (left picture) and CX3CL1 (right picture), (B) CD11b+ cells (left picture) and CX3CL1 (right picture), (C) Ly6G+ cells (left picture) and CX3CL1 (right picture), (D) CD4+ cells (left picture) and CX3CL1 (right picture), (E) CD8a+ cells (left picture) and CX3CL1 (right picture), and (F) FB (fibroblast)-marker+ cells (left picture) and CX3CL1 (right picture) using a 200x magnification. Arrows highlight overlapping areas. WT = wild-type; CVB3 = Coxsackievirus B3.
(TIF)

S2 Fig Regulation of chemokine receptors and the corresponding chemokines in the spleen of Coxsackievirus B3-infected mice. Bar graphs represent the mean ± SEM of splenic (A) CCR2, (B) MCP-1, (C) CX3CR1, and (D) CX3CL1 gene expression, as indicated, after normalization to the housekeeping gene 18S using the 2−ΔCt formula and normalized to the WT group, which was set as 1. Statistical analysis was performed by One-way ANOVA or the Kruskal-Wallis test. *p<0.05, **p<0.01, ***p<0.001 with n = 7–12 per group.
(TIF)
