# Supplementary figures and images for: CX3CR1 knockout aggravates Coxsackievirus B3-induced myocarditis
Source: PLoS One. 2017 Aug 11;12(8):e0182643. doi: 10.1371/journal.pone.0182643 (PMC5553786; doi:10.1371/journal.pone.0182643)

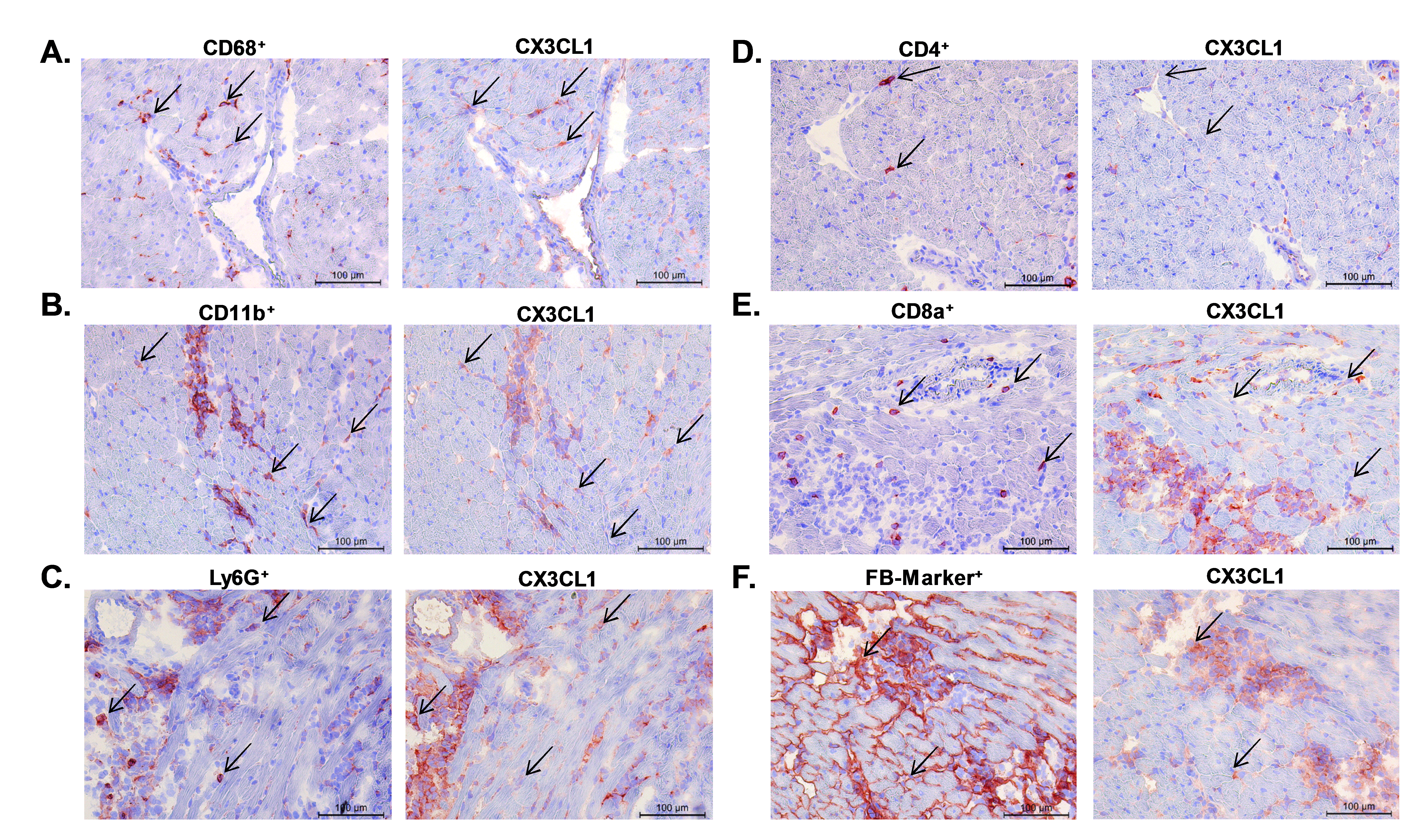

Supplement: S1 Fig — Representative pictures of immunohistological stainings on successive tissue samples from LV (A) CD68+ cells (left picture) and CX3CL1 (right picture), (B) CD11b+ cells (left picture) and CX3CL1 (right picture), (C) Ly6G+ cells (left picture) and CX3CL1 (right picture), (D) CD4+ cells (left picture) and CX3CL1 (right picture), (E) CD8a+ cells (left picture) and CX3CL1 (right picture), and (F) FB (fibroblast)-marker+ cells (left picture) and CX3CL1 (right picture) using a 200x magnification. Arrows highlight overlapping areas. WT = wild-type; CVB3 = Coxsackievirus B3. (TIF) [file pone.0182643.s001.tif]

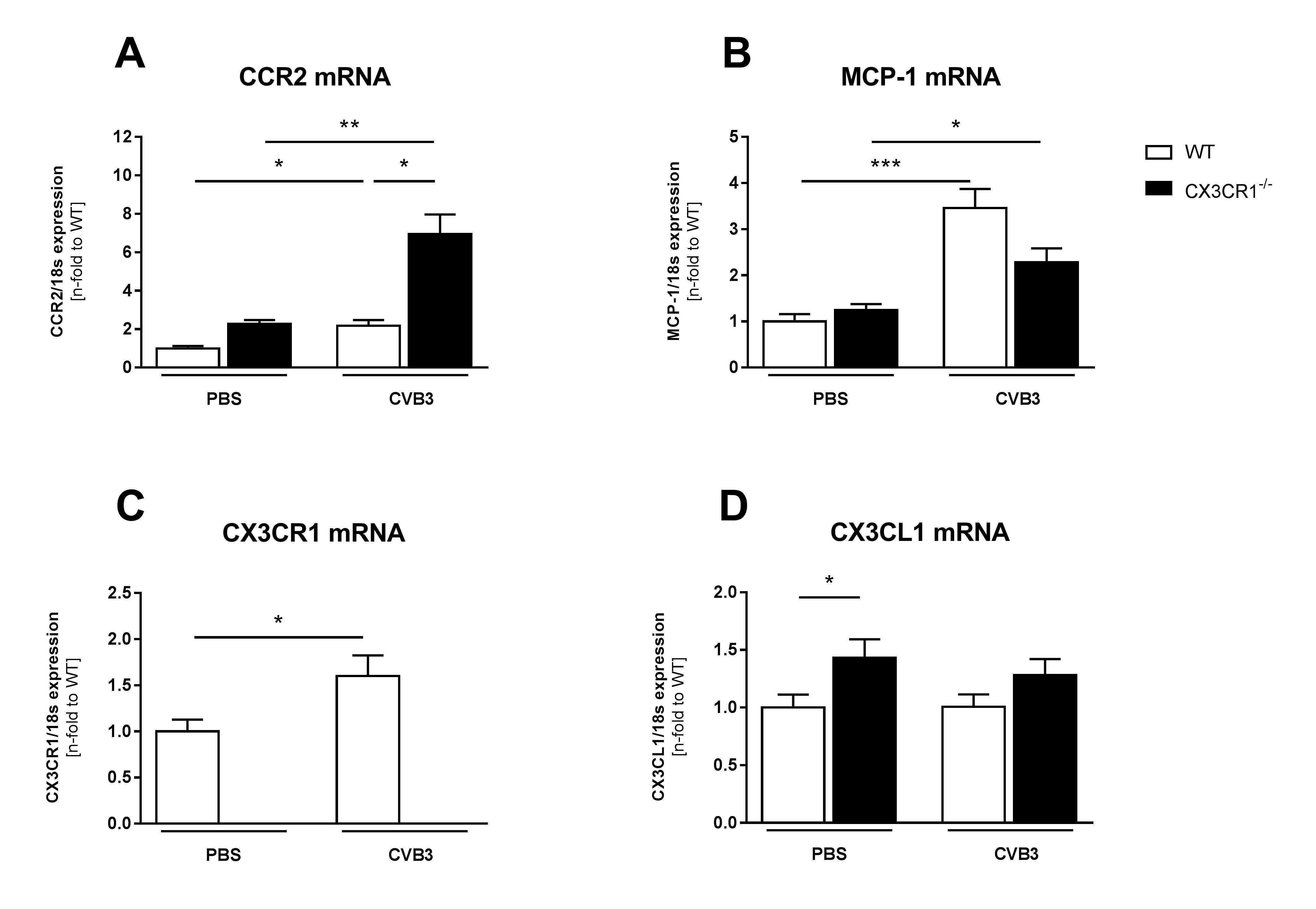

Supplement: S2 Fig — Bar graphs represent the mean ± SEM of splenic (A) CCR2, (B) MCP-1, (C) CX3CR1, and (D) CX3CL1 gene expression, as indicated, after normalization to the housekeeping gene 18S using the 2−ΔCt formula and normalized to the WT group, which was set as 1. Statistical analysis was performed by One-way ANOVA or the Kruskal-Wallis test. *p<0.05, **p<0.01, ***p<0.001 with n = 7–12 per group. (TIF) [file pone.0182643.s002.tif]
